# Enhanced Cellulose Degradation Using Cellulase-Nanosphere Complexes

**DOI:** 10.1371/journal.pone.0042116

**Published:** 2012-08-01

**Authors:** Craig Blanchette, Catherine I. Lacayo, Nicholas O. Fischer, Mona Hwang, Michael P. Thelen

**Affiliations:** Physical and Life Sciences, Lawrence Livermore National Laboratory, Livermore, California; Consejo Superior de Investigaciones Cientificas, Spain

## Abstract

Enzyme catalyzed conversion of plant biomass to sugars is an inherently inefficient process, and one of the major factors limiting economical biofuel production. This is due to the physical barrier presented by polymers in plant cell walls, including semi-crystalline cellulose, to soluble enzyme accessibility. In contrast to the enzymes currently used in industry, bacterial cellulosomes organize cellulases and other proteins in a scaffold structure, and are highly efficient in degrading cellulose. To mimic this clustered assembly of enzymes, we conjugated cellulase obtained from *Trichoderma viride* to polystyrene nanospheres (cellulase:NS) and tested the hydrolytic activity of this complex on cellulose substrates from purified and natural sources. Cellulase:NS and free cellulase were equally active on soluble carboxymethyl cellulose (CMC); however, the complexed enzyme displayed a higher affinity in its action on microcrystalline cellulose. Similarly, we found that the cellulase:NS complex was more efficient in degrading natural cellulose structures in the thickened walls of cultured wood cells. These results suggest that nanoparticle-bound enzymes can improve catalytic efficiency on physically intractable substrates. We discuss the potential for further enhancement of cellulose degradation by physically clustering combinations of different glycosyl hydrolase enzymes, and applications for using cellulase:NS complexes in biofuel production.

## Introduction

The inefficient conversion of plant-derived cellulose to fermentative sugars has been identified as one of the limiting factors for widespread production of alternative fuels from lignocellulose feedstocks [1]. New methods to increase cellulase enzyme kinetics and stability are critical for the economic feasibility of biofuel production. Most current industrial processing of plant biomass involves harsh thermochemical pretreatment to open up the physical structure of intricately complexed polymers of lignin, cellulose and hemicellulose polysaccharides, followed by hydrolysis of the cellulose using microbial enzymes that are free in solution. In contrast, particulate enzymes that are highly effective in deconstructing untreated biomass are found in bacterial cellulosomes. These multiprotein complexes assemble different glycosyl hydrolases on a scaffold protein [1]–[3], promoting synergy and increased efficiency in cellulolytic action [4], [5]. This cooperation of physically associated enzymes has lead to the notion of using synthetic biology to engineer novel cellulosomes for lignocellulose breakdown [4]–[6]. However, bacterial assembly of the cellulosome has been shown to occur through a tightly controlled and complex process, and little is known about the essential factors in this process [7]. Thus, the production of engineered cellulosomes for use in biofuel processing remains elusive.

Since the key feature of the enzymatic efficiency of cellulosomes is the clustering of cellulases in a single macromolecular complex, we hypothesize that assembling an isolated cellulase onto a suitable synthetic nano-scale material could result in an increase in enzymatic efficiency. A robust nano-scale platform, such as polymeric nanoparticles, can serve as an analog to the cellulosomal scaffold that holds together individual enzymes in the multimeric complex. This approach can be used with cellulase enzymes that have already been purified and extensively characterized, therefore bypassing the difficulties in recombinant methods required to engineer cellulosomes. To test our idea, we conjugated a cellulase to spherical nanometer-size beads (nanospheres), and characterized the enzymatic activity of the cellulase-nanosphere complex (cellulase:NS) on different substrates: soluble carboxymethyl cellulose (CMC); insoluble microcrystalline cellulose; and cellulose thickenings in secondary cell walls of cultivated wood cells [8]. We demonstrate that clustering the cellulase on nanospheres results in significant enhancement of enzyme efficiency on insoluble substrates due to increasing enzyme-substrate interactions, and we consider the potential for further applications of the cellulase:NS approach in biofuel processing.

## Results

### Cellulases can be covalently immobilized on nanoparticles

**Figure 1:**
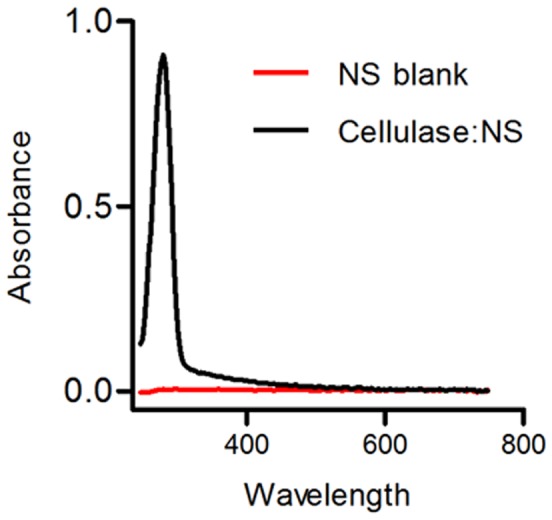
Absorbance spectrum of cellulase:NS complex. The spectrum of the cellulase:NS complex (black line) was captured after blanking the instrument with NS (red line) that had not been conjugated to cellulase enzymes. The clear peak at 280 nm and lack of additional peaks at absorbance wavelengths higher than 320 nm indicates that there was no interference in the readings from the nanospheres.

To mimic the characteristic clustered presentation of cellulases on bacterial cellulosomes, an endoglucanase from *Trichoderma viride* was covalently immobilized on 20 nm diameter polystyrene nanospheres. Using carboxyl-functionalized nanospheres, the cellulase was readily conjugated using traditional 1-ethyl-3-(3-dimethylaminopropyl) carbodiimide hydrochloride (EDC)/*N*-hydroxysulfo-succinimide (Sulfo-NHS) chemical crosslinking. Successful conjugation of enzyme to the nanospheres was assessed by spin filtration, where cellulase was easily tracked by 280 nm absorbance, or simply by color. Free cellulase passed through the 300 kDa molecular weight cut off (MWCO) membrane, whereas nanospheres and cellulase:NS were retained, confirming successful conjugation. The concentration of protein bound to NS was calculated by comparing the absorbance at 280 nm with the absorbance of the stock cellulase solution (50 mg/ml). Since the nanospheres displayed some intrinsic absorbance between 230 and 400 nm (including at 280 nm), this was subtracted from samples by using a solution of NS that were treated exactly as the test samples except for the addition of enzyme. The spectra of the cellulase:NS and blank unconjugated NS are shown in Figure 1. A single protein peak was observed in the cellulase:NS spectrum in the aromatic region (260–300 nm wavelength) with no absorbance above 320 nm, indicating that there was no interference from the NS during these measurements. The cellulase concentration in the cellulase:NS complex was also reproducibly calculated at various dilutions using this approach.

### Immobilized cellulase exhibits enhanced activity on insoluble cellulose

**Figure 2:**
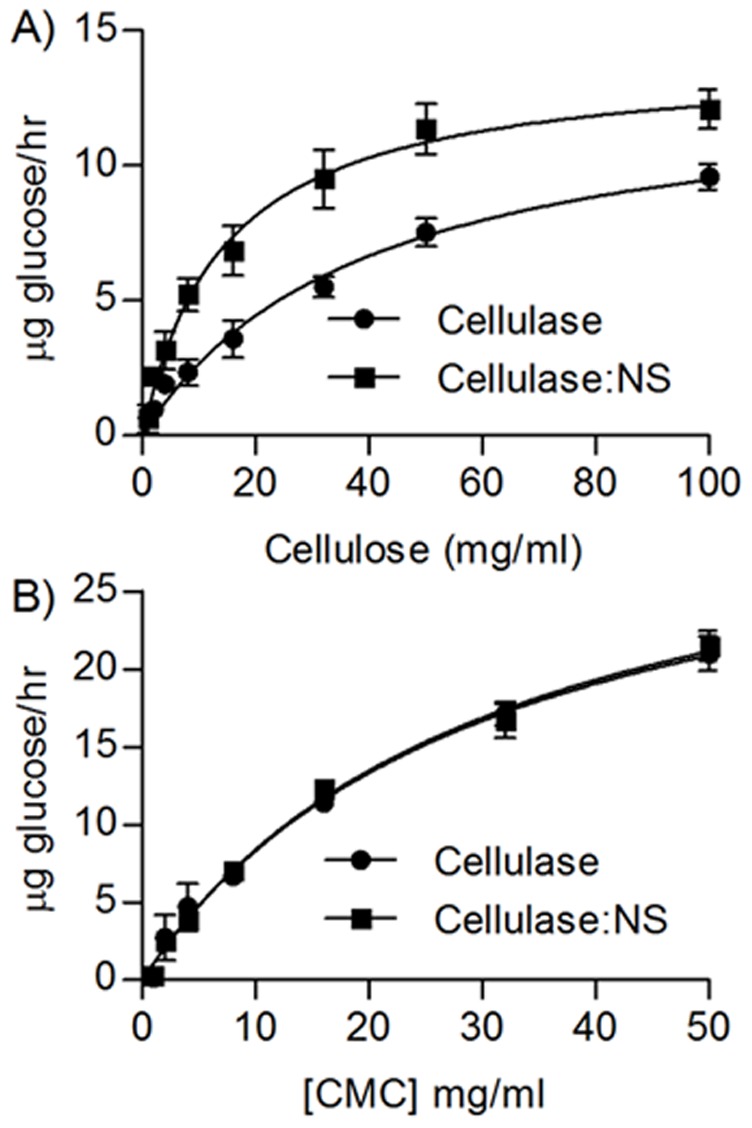
Comparison of cellulase and cellulase:NS enzyme kinetics. The velocity of glucose release is plotted as a function of A) insoluble cellulose concentration, and B) CMC concentration. Open circles, cellulase; black circles, cellulase-bead complex.

To investigate the effect of complexing multiple cellulase copies on a synthetic scaffold, cellulase and cellulase:NS reaction kinetics were compared. The release of glucose was measured over a range of substrate concentrations, using microcrystalline cellulose and soluble CMC. CMC is a derivative form of cellulose, chemically modified with carboxylmethyl groups (-CH2-COOH) at the hydroxyl moiety of glucopyranose units. Since this significantly improves the solubility of cellulose in aqueous buffers, CMC was used to evaluate the effects of substrate solubility on the cellulase:NS activity. For insoluble cellulose, approximately 50–130% more glucose was liberated by cellulase:NS relative to cellulase alone at the concentrations tested (1–100 mg/ml) (Figure 2A). Cellulose hydrolysis by both immobilized and free cellulases followed Michaelis-Menten kinetics, and rate constants K_m_ and V_max_ were calculated (Table 1). When microcrystalline cellulose was used as a substrate, the V_max_ for the cellulase:NS complex (13.9 µg glucose/hr) was comparable to the V_max_ for the cellulase alone (13.2 µg glucose/hr). However, the K_m_ for the cellulase:NS complex (14.3 mg/ml) was more than 2-fold lower than the K_m_ for cellulase alone (39.5 mg/ml), indicating that the cellulase:NS complex had a significantly higher affinity for microcrystalline cellulose. When using CMC, no significant difference was observed between free cellulase and cellulase:NS reactions at the substrate concentrations tested (1–50 mg/ml). Under these conditions, the reaction rates at all substrate concentrations were identical between the two samples (Figure 2B). Consistent with this, no differences in the K_m_ (30.2 and 30.5 mg/ml) and V_max_ (33.5 and 34.0 µg/hr) were found between free cellulase and cellulase:NS complex, respectively. It is worth noting that CMC substrate concentrations higher than 50 mg/ml could not be tested due to experimental limitations. At 100 mg/ml, the CMC solution was extremely viscous and the measured reaction rate decreased significantly. These results indicated that the enzymatic reaction in high viscosity solution was diffusion limited and could not be compared with or analyzed in the same manner as the reactions at lower CMC concentrations. Therefore, complete saturation was not reached for CMC, which likely decreased the overall accuracy of the calculated Vmax values. However, at all conditions tested for CMC, no difference was observed between the cellulase and cellulase:NS constructs, demonstrating that conjugation to NS had little effect on enhancing the activity of the enzyme.

**Table 1:** Kinetic parameters of glucose release from cellulose and CMC substrates using cellulase: NS complex compared to free cellulase enzyme.

Sample	Substrate	V_max_ (µg glucose/hr)	K_m_ (mg/ml)
Cellulase	Cellulose	13.2+/−1.1	39.5+/−7.1
Cellulase:NS	Cellulose	13.9+/−0.5	14.3+/−1.6
Cellulase	CMC	33.5+/−2.6	30.2+/−4.7
Cellulase:NS	CMC	34.0+/−2.5	30.5+/−4.5

Values are the mean +/− standard deviation.

### Cellulase:NS digests Zinnia elegans wood cells more efficiently than free cellulase

Because cellulase:NS constructs performed better than the free enzyme on purified crystalline cellulose, we also examined their activities on a substrate more representative of actual biomass. Cultured wood cells from *Zinnia elegans* [8]–[10] were chosen as substrates because they are a homogeneous source of lignocellulosic material, especially when compared to plant stems or traditional biomass. The patterned deposits of secondary wall in *Zinnia* xylem cells consist primarily of cellulose microfibrils, hemicelluloses, and lignin, forming a structure that is consistent with the composition observed in natural substrates for biofuel production. Plant cell walls provide a water resistant and mechanically robust structure due to the presence of a multitude of different polysaccharides, phenolic compounds, and proteins, which are also represented in these cells. In addition, methods have been developed to isolate homogeneous (>90%) populations of wood cells, greatly facilitating the analysis of the structure, composition, and degradation of the cell wall [8] when compared to plant tissues containing multiple cell types that differ in lignocellulose content.

**Figure 3:**
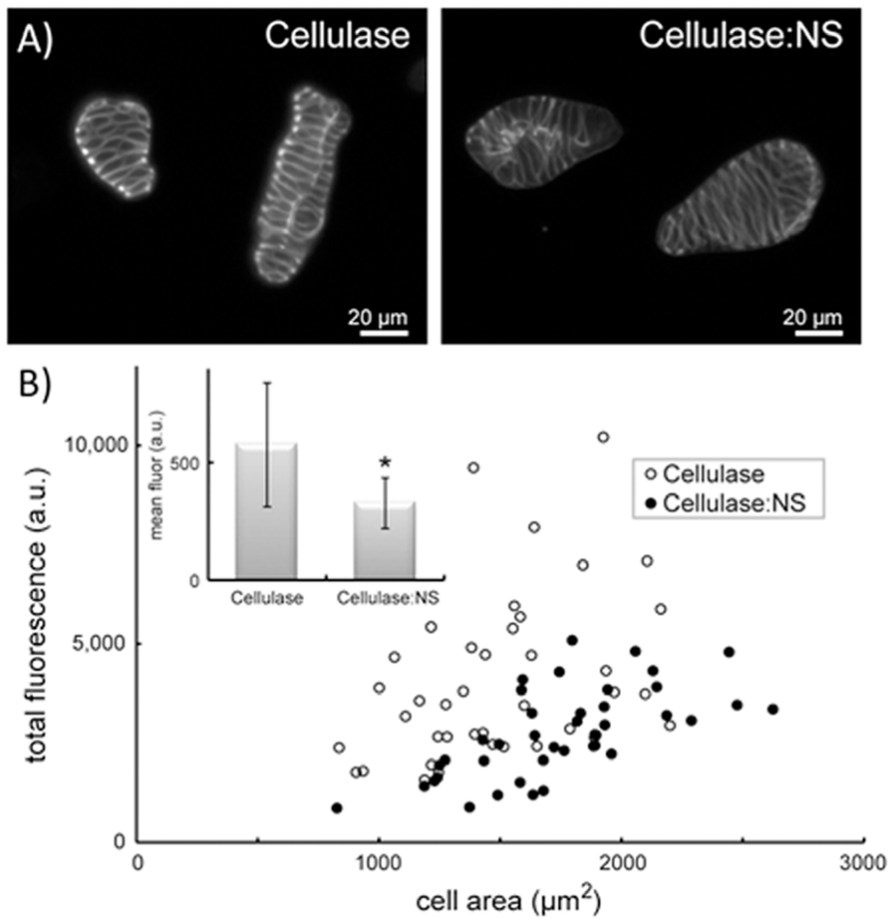
Efficiency of cellulose removal from wood cells by cellulase:NS. A) A cellulose specific probe (*Ct*CBM3-GFP) was used to fluorescently label cultured wood cells digested with either free cellulase (left) or cellulase:NS (right). Scale bar = 20 µm. B) The total fluorescence of individual wood cells was quantified and plotted as a function of cell area after degradation with cellulase (open circles) or cellulase-NS (closed circles). Inset shows the mean fluorescent intensity of each population of digested cells (n = 40), which are significantly different (*p<0.0001, Mann-Whitney test). Error bars = SD.

Prior to the enzymatic digest using free cellulase or cellulase:NS, wood cells were incubated in hot acidified chlorite, which has been shown to delignify cell walls and improve the hydrolysis of biomass by cellulolytic enzymes [8], [11], [12]. *Zinnia* wood cells were incubated with free cellulase or cellulase:NS under the same reaction conditions used with the synthetic substrates; however, since the measurement of glucose released in the wood cell reactions is below the limit of detection in our assay, we used a novel method to quantitate cell wall cellulose [8]. The amount of cellulose remaining after digestion was measured using a fluorescent probe that specifically binds to crystalline cellulose: a recombinant protein consisting of a carbohydrate-binding module from *Clostridium thermocellum* fused to green fluorescence protein (*Ct*CBM3-GFP) [13]. Cells subjected to cellulase treatments were washed to remove cellulase, incubated with *Ct*CBM3-GFP, and subsequently imaged by fluorescent microscopy (Figure 3A). Single cell fluorescence intensities were quantified for both free cellulase and cellulase:NS reactions, and statistical population analysis was performed (Figure 3B). Using this method, we determined that the mean fluorescence intensity was 43% lower in wood cell populations digested with the cellulase:NS complex relative to free cellulase, indicating that more cellulase hydrolysis had occurred than in the free cellulase reaction. These results confirm those observed with microcrystalline cellulose, and demonstrate that complexing cellulase onto a nanoparticle scaffold enhances its overall enzymatic activity on a biologically-relevant substrate.

## Discussion

**Figure 4:**
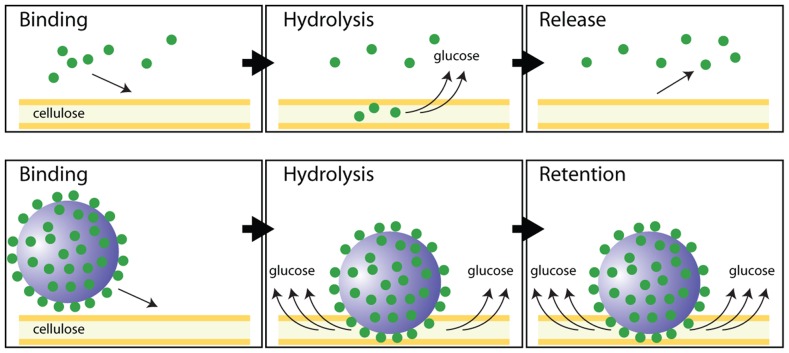
Schematic diagram of enhanced glucose release from a crystalline cellulose substrate using the cellulase:NS construct relative to free cellulase.

A potential approach to improving the deconstruction of cellulosic biomass in the initial steps of biofuel production is to mimic highly efficient cellulosome enzymatic complexes. A key feature of cellulosomes is the clustering of glycosyl-hydrolase enzymes in a single macromolecular complex, which we attempted to simply replicate by conjugating a single type of cellulase enzyme to an artificial scaffold of roughly the same size as the cellulosomal scaffold. When we immobilized cellulase to 20 nm polystyrene nanospheres, the cellulase:NS complex exhibited significantly enhanced enzymatic degradation of insoluble cellulose relative to free cellulase. No differences were observed between bound and free enzymes using a soluble cellulose substrate (CMC), even though the overall activity by both cellulases was higher than on the insoluble substrate (Figure 2). This effect can be attributed to an increase in the overall affinity of the cellulase:NS complex to the microcrystalline cellulose substrate through an avidity effect, which results from multiple interactions between the enzyme and substrate. By increasing local retention of the cellulase:NS complex on insoluble cellulose due to multiple simultaneous interactions between immobilized cellulase and the insoluble substrate, the effective enzyme concentration is increased compared to free cellulases, which are dependent on diffusion parameters. A schematic of this proposed mechanism is provided in Figure 4.

Similar multivalent interactions have been widely exploited to enhance binding between low affinity ligands and their receptors [14], [15]. Furthermore, clustering of multiple ligands has been shown to enhance the avidity as well as recognition specificity of weak interactions between carbohydrates and their protein partners [16]–[18] and has been shown to increase the biological activity of weakly binding therapeutics [19]. Thus, we believe the increase in overall activity on insoluble cellulose by cellulase:NS is a direct result of the enhanced affinity between the clustered enzyme and cellulose due to multivalent interactions. Accordingly, no enhancement of cellulase:NS activity was observed when the soluble CMC substrate was used. At the substrate and enzyme concentrations used in these experiments (1–50 mg/mL and 0.1 mg/mL, respectively), diffusion of soluble substrate (CMC) ensures continuous availability to cellulase regardless of immobilization. Under these conditions, there is no enhanced affinity of the cellulase:NS complex with substrate. In contrast, access to insoluble substrates (microcrystalline cellulose and wood cell secondary wall cellulose) is not dictated by free diffusion; consequently, the increased affinity due to clustering of the cellulase enzyme leads to significantly enhanced enzymatic activity.

It is also worth noting that the Michaelis constants calculated in this manuscript were higher than the values reported in some previous studies. Imai et al [20], reported K_m_ values ranging from 3–11 mg/ml and Liu et al [21] reported a K_m_ value of 0.9 mg/ml, whereas we calculated K_m_ values of 14.3–39.5 mg/ml and 30.2–30.5 mg/ml for the cellulose and CMC substrates, respectively. These discrepancies are likely due to differences in the experimental approaches used. Imai et al [20] examined the effects of subjecting the substrate to ultrasonication treatment on the kinetics of cellulose degradation by *T. viride* cellulase enzymes and the substrate used was a CMC derivative that contained a very low degree of carboxylmethyl substitution and was still insoluble in aqueous buffer. Therefore, the lower K_m_ values reported in this work were likely due to a combination of the ultrasonication treatment and difference in substrate. Furthermore, in the study by Liu et al [21], a bifunctional enzyme displaying both chitosanase and CMC cellulase activity was purified form a commercially available *T. viride* cellulase complex mixture and the Michaelis-Menton kinetics were only evaluated using the purified enzyme. Thus, the significantly lower K_m_ value reported in this work was likely due to the fact that only a single purified enzyme was analyzed, whereas in the present study we measured the kinetics of CMC degradation using the unpurified *T. viride* material.

Covalent immobilization of enzymes to substrate surfaces has been shown in some cases to result in reduced activity due to the potential binding of key functional groups positioned within or near the active site [22]. In a previous study, cellulase immobilized on polyvinyl alcohol coated chitosan beads retained only 87% relative activity using CMC as a cellulose substrate [23]. Cellulase immobilized on magnetite nanoparticles exhibited full retention of activity only after considerable optimization of conjugation parameters was conducted [24]. The conclusion from these studies is that most cellulase enzymes retain their function after immobilization under proper conjugation conditions. In these previous studies, the potential effect of enzyme clustering on enzymatic activity was not considered and the immobilized enzyme clusters were assumed to have the same activity as the free cellulose enzyme. In our study, glucose release was identical between free cellulase and the cellulase:NS complex when CMC was used as the substrate. Based on that, we could conclude that cellulase enzymes retain their activity after conjugation. However, based on the synergistic effects observed when insoluble cellulose was used as a substrate, this does not appear to be a valid conclusion. It follows that the calculated relative activity should be considered an upper limit, since a synergistic effect may actually enhance the enzymatic activity of clustered enzymes, which would artificially increase the calculated relative activity.

Although our results from using artificial substrates pointed to an enhancement in cellulase:NS activity on cellulose, we wanted to determine whether this could be relevant to biomass processing. Most cellulase studies are carried out on one of these two cellulose substrates because they are pure and easy to obtain commercially, but these experiments could be misleading for the application of cellulases to plant biomass. For example, an enzyme that is active on a soluble cellulose derivative may have low or no activity on an insoluble substrate. Likewise, when calculating enzyme efficiencies and comparing kinetics, microcrystalline cellulose may give different results compared to the natural cellulose found in biofuel feedstocks. To provide a natural substrate comparison, we used cultured plant cells because their walls can be more effectively manipulated *in vitro* using chemical and enzymatic treatments compared to tissue [25]. More specifically, we used a well-established system of cultured wood cells from *Zinnia elegans*, which has proven to be an ideal system to examine woody tissue formation *in vitro* [9], [10] and is amenable to chemical manipulation, microscopy, and cell population analysis [8], [26]–[28]. This system generates synchronous cell cultures, which consist of a single cell type at the same developmental stage, greatly facilitating the analysis of structure, composition and degradation of the cell wall when compared to plant tissue containing heterogeneous cell populations. When fully differentiated, *Zinnia* cells are suitable single cell biomass analogs because they contain highly organized secondary wall deposits that represent the majority of lingocellulose found in trees and most plants. Here, we found that compared with free cellulase, cellulase:NS removes a significantly higher amount of cellulose, corroborating almost exactly the overall activity and kinetics of cellulase:NS *vs* cellulase action that we measured using the microcrystalline cellulose substrate. From these studies, we suggest that cultured wood cells offer an excellent model system, not only for cellulase activity, but perhaps also to quantitatively assess the action of glycosyl-hydrolases, lignin-specific enzymes, and chemical treatments that could accelerate research towards more efficient biofuel production.

## Conclusions

Biofuel production from lignocellulosic biomass is critically dependent on the enzyme-catalyzed hydrolysis of cellulose, the major constituent of plant feedstocks. One of the most significant bottlenecks in this process is the low efficiency of cellulase enzymes used to catalyze cellulose degradation. While the utilization of native enzymatic complexes, such as bacterial cellulosomes, represents a potential approach to overcome this barrier, the difficulty of biological mass-production precludes their near-term use for routine hydrolysis of biomass. However, the use of engineered cellulosomes will have two very distinct advantages: 1) different glycosyl hydrolases can be placed in close proximity to provide synergistic action on lignocellulosic substrates; and 2) enzymes from different species that have superior activities on a given substrate can be assembled into a single complex specifically designed for each type of substrate. Therefore, in this study we designed synthetic enzyme complexes that mimic the clustered organization of enzymes in cellulosomes by immobilizing a commercially available cellulase to nanoparticles. We found these cellulase:NS complexes more efficiently degraded microcrystalline cellulose with a significantly higher affinity relative to free cellulase. Similarly, the activity of cellulase:NS resulted in greater removal of cellulose from cultured wood cells relative to free cellulase, indicating that the concept of an engineered cellulosome will function on a natural lignocellulosic substrate related to feedstock biomass. The combined results of this study point to a new technological approach that could improve the enzymatic degradation of lignocellulosic material, and perhaps stimulate the activity and affinity of other enzymes to insoluble substrates such as lignin. In addition, it is likely that further enhancement of cellulose degradation in biofuel feedstocks would be realized by immobilizing different cellulases and combinations of cellulase, xylanase, and laccase/lignase enzymes to nanoparticle scaffolds.

## Materials and Methods

### Conjugation of cellulase to polystyrene nanospheres

Cellulase was conjugated to 20 nm carboxyl-terminated polystyrene nanospheres (Bangs Laboratory; Fishers, IN) using 1-ethyl-3-(3-dimethylaminopropyl) carbodiimide hydrochloride (EDC) and *N*-hydroxysulfosuccinimide (Sulfo-NHS) coupling chemistry [29]. For conjugation, 20 µl of the nanosphere stock solution (∼2×10^10^ nanospheres/ml) was diluted to a final volume of 1 ml in Milli-Q water. The solution was transferred to a centrifugal concentrator with a molecular cutoff of 300 kDa (Vivaspin 500) and centrifuged at 15,000 rcf for 5 min. The nanospheres were then re-suspended in 0.5 ml of 0.1 M MES buffer (pH 4.7) containing 0.8% NaCl, and transferred to a microcentrifuge tube. EDC (1 mg) and Sulfo-NHS (1 mg) were then added, and the solution incubated at room temperature with gentle mixing for 20 min. The solution was then transferred to a centrifugal concentrator with a molecular weight cutoff (MWCO) of 300 kDa (Vivaspin 500) and centrifuged at 15,000 rcf for 5 minutes. After a second centrifugation step, the solution was re-suspended in 10 mM sodium phosphate buffer containing 150 mM NaCl at pH 7.4 (PBS), and again transferred to a microcentrifuge tube. Subsequently, 50 mg of cellulase from *Trichoderma viride* 1,4-(1,3;1,4)-β-D-Glucan 4-glucano-hydrolase, Onozuka RS, EC number 3.2.1.4 (Sigma-Aldrich) was dissolved in 1 ml of 50 mM sodium citrate buffer, pH 5.0. The cellulase solution (200 µl, 50 mg/ml) was then added to the functionalized nanospheres. The cellulase-nanosphere (cellulase:NS) mixture was incubated at room temperature in a tube rotator for 2 h followed by three wash steps with 50 mM sodium citrate buffer using the centrifugal concentrators as described above to remove unbound protein. The concentration of protein bound to the nanospheres was calculated by comparing the absorbance at 280 nm with the absorbance of the stock cellulase solution (50 mg/ml). Since the nanospheres displayed some intrinsic absorbance at 280 nm, readings were corrected using a solution of nanospheres that went through the same process described above except without the addition of the cellulase enzyme. The cellulase concentration in the cellulase:NS complex was calculated reproducibly at various dilutions using this approach. During the centrifugal concentration steps, cellulase:NS preparations were tracked based on the brownish color of the *T. viride* cellulase (Figure 1) as well as by A_280_ measurements.

### Free cellulase and cellulase:NS activity assays

Glucose release during digestion of both microcrystalline cellulose and CMC (Sigma-Aldrich) was measured using the colorimetric dinitrosalicyclic acid (DNS) sugar reducing assay [30]–[32]. The assay measurements were carried out at substrate concentrations ranging from 1 to 100 mg/ml for insoluble cellulose and 1 to 50 mg/ml for soluble CMC. In these experiments, free cellulase or cellulase:NS complex was added to the substrate at a final concentration of 0.1 mg/ml in 300 µl, and reactions were carried out at 37°C and pH 5.5 with gentle agitation for 5.5 h. After removing the tubes from the incubator, reactions were centrifuged at 5000 rcf for 5 min and the supernatant containing released glucose was analyzed. For the soluble CMC substrate reactions, samples were analyzed immediately, whereas the particulate cellulose reaction supernatants were removed and stored at 4°C until analysis. Glucose release was analyzed by adding 150 µl of the DNS reagent to 50 µl of the sample. The mixture was then boiled for 5 min and 100 µl was transferred to 96 well plates. The absorbance of each sample was read at 540 nm using a spectrophotometer plate reader (Biotek MultiMode. All reactions were performed in triplicate and each plate included a standard curve with glucose concentrations ranging from 25–400 µg/ml. Kinetic parameters of the reaction (K_m_ and V_max_) were then calculated by fitting the data (reaction rate (*v*) vs substrate concentration [S]) to the equation, *v* = (V_max_*[S]/(K_m_+[S]), derived from Michaelis-Menten kinetics. Data fits were achieved using ordinary least squares models in IGOR Pro 6.0 software.

### Preparation of cultured wood cells

*Zinnia elegans* leaf mesophyll cells were differentiated in culture for 8 days followed by density gradient separation of wood cells (tracheary elements) from dead and undifferentiated cells as described previously [8]. Percoll fractions containing approximately 90% wood cells were washed three times with distilled, deionized water by low speed centrifugation. Cells were delignified by incubation in acidified chlorite (1% sodium chlorite, 0.14% acetic acid) at 70°C [33] for 20 h and then washed three times with water as before. Lignin removal by this oxidative treatment was confirmed by the loss of cell wall autofluorescence and staining with the lignin dye phloroglucinol.

### Enzymatic digest of wood cells and cellulose quantification

Approximately 25 mg/ml of delignified wood cells (based on the wet weight of the cell pellet) were incubated with cellulase enzymes at a concentration of 0.1 mg/ml under the same reaction conditions described above. After incubation, cells were washed three times by low speed centrifugation in PBS containing 1% BSA, (PBS-BSA). Cell wall cellulose was fluorescently labeled by incubation with 0.1 mg/ml GFP-tagged CBM3 from *Clostridium thermocellum* (*Ct*CBM3-GFP) [13] in PBS-BSA for 1.5 h at room temperature with gentle mixing. Cells were finally prepared for fluorescence microscopy by washing three times with PBS-BSA. Cell population analysis for quantitation of cellulose fluorescence following *Ct*CBM3-GFP labeling was performed as previously described [8].
